# Parathyroid Hormone Reduction Predicts Transient Hypocalcemia after Total Thyroidectomy: A Single-Center Prospective Study

**DOI:** 10.1155/2020/7189857

**Published:** 2020-08-01

**Authors:** Kangnan Mo, Jinbiao Shang, Kejing Wang, Jialei Gu, Peng Wang, Xilin Nie, Wendong Wang

**Affiliations:** ^1^Department of Head and Neck Surgery, Cancer Hospital of University of Chinese Academy of Sciences (Zhejiang Cancer Hospital), No. 1 Banshan Road (East), Gongshu, Hangzhou 310022, China; ^2^Department of Head and Neck Surgery, Institute of Cancer and Basic Medicine (IBMC), Chinese Academy of Sciences, No. 1 Banshan Road (East), Gongshu, Hangzhou 310022, China

## Abstract

**Objective:**

We performed this study to investigate the risk factors for postoperative hypocalcemia after total thyroidectomy with central lymph node dissection (CLND). *Study Design*. This was a single-center prospective study based on 176 consecutive patients who underwent total thyroidectomy for papillary thyroid carcinoma. *Setting*. Patients were recruited between January 2016 and June 2018. *Subjects and Methods*. Patients who underwent bilateral (*n* = 155, bilateral group) and ipsilateral CLND (*n* = 21) after total thyroidectomy were included. The preoperative and postoperative parathyroid hormone (PTH) and calcium levels were detected. The risk factors for transient hypocalcemia were identified using logistic regression analysis and receiver operating characteristic (ROC) curve analysis.

**Results:**

Fifty-one (28.98%) patients developed transient hypocalcemia, and 2 patients (1.14%) developed permanent hypoparathyroidism. There was no difference in the gender ratio or the morbidity of hypocalcemia between the patients who underwent bilateral and ipsilateral CLND. On postoperative day 1, PTH decrease was a risk factor for transient hypocalcemia in the whole cohort (*β* = 0.043, OR = 1.044, 95% CI 1.023–1.065, *p* < 0.001), bilateral group (*β* = 0.042, OR = 1.043, 95% CI 1.022–1.064, *p* < 0.001), and female patients (*β* = 0.049, OR = 1.050, 95% CI 1.026–1.075, *p* < 0.001). Tumor diameter was a risk factor for transient hypocalcemia in female patients (*β* = 0.499, OR = 1.647, 95% CI 1.003–2.704, *p*=0.049). The ROC curve analysis illustrated that 65.58%, 71.00%, and 71.00% PTH level reduction had high accuracy in predicting transient hypocalcemia in the whole cohort, bilateral group, and female patients, respectively (AUC = 0.986, 0.987, and 0.987).

**Conclusion:**

Asymptomatic female patients with bilateral CLND and a 71.00% PTH level reduction were at a high risk of transient hypocalcemia.

## 1. Introduction

Total thyroidectomy or radical thyroidectomy is the main surgical management for papillary thyroid carcinoma (PTC). Transient postoperative hypocalcemia is a common complication following thyroidectomy with a worldwide incidence of 3∼30% [1–3]. Female gender is a risk factor for transient hypocalcemia. Approximately 25∼40% of female patients developed temporary postoperative hypocalcemia, which is higher than the ∼10% in male patients [1, 3]. Although the recurrence of secondary hypoparathyroidism, especially in chronic kidney disease could be significantly reduced by autotransplantation [4, 5], the high incidence of transient hypocalcemia after total thyroidectomy is still a dilemma.

The treatment strategy for PTC remains controversial due to the incidence of postoperative hypoparathyroidism and hypocalcemia. Total thyroidectomy plus central lymph node dissection (CLND) potentially reduces the recurrence of PTC compared to total thyroidectomy alone [6, 7]. However, several trials demonstrated that the combination of bilateral or ipsilateral CLND could increase the incidence of transient or permanent hypocalcemia and prolong the length of hospital stay [7, 8]. In addition, the malignant pathology, age, female gender, and number of parathyroid glands are indicated as risk factors for asymptomatic and transient hypocalcemia [8, 9].

The postoperative serum calcium (Ca) level decreased up to five days postthyroidectomy, and the late measurement of Ca levels lacked the sensitivity to predict hypocalcemia. Many surgeons are interested in predicting early hypocalcemia posttotal thyroidectomy and, therefore, use the intraoperative parathyroid hormone (PTH) and postoperative 1-hour or 4-hour PTH level test [2, 10, 11]. Surgeons reported that the postoperative 1-hour PTH level is reliable for predicting the development of postoperative symptomatic hypocalcemia [2, 11, 12]. Reddy et al. reported that the PTH level at 20 minutes after total thyroidectomy was reliable for predicting clinical hypocalcemia [12]. Surgeons discovered that the postoperative 1-hour PTH level had equivalent reliability to the postoperative day 1 PTH for predicting the development of symptomatic hypocalcemia [11]. Seo et al. evaluated the postoperative 1-hour PTH and postoperative days 1, 3, 5, and 7 PTH levels and found that the predictive power of the postoperative 1-hour PTH was equivalent to that of postoperative day 1 [13].

Other surgeons suggested that the decrease in PTH levels was more precise than the postoperative PTH [14]. Lecerf et al. suggested the decrease in the PTH level (68.5%) was more precise than the postoperative PTH level (19.4 ng/l) for predicting hypocalcemia after total thyroidectomy, and the patients with a reduction in PTH of less than 68.5% could be discharged on postoperative day 1 [14]. Updated reports on the postoperative PTH levels for predicting hypocalcemia increased pressure for early discharge of patients and freed patients from unnecessary Ca supplements.

We performed this study to investigate the difference in risk factors between patients with dissimilar surgical strategies and in female patients. The association of age, gender, preoperative PTH level, Ca level, and PTH level reduction on postoperative day 1 with the incidence of postoperative hypocalcemia after total thyroidectomy with CLND was identified. We aimed to determine whether these variables were risk factors for hypocalcemia in patients after total thyroidectomy.

## 2. Materials and Methods

### 2.1. Setting and Subjects

This was a single-center prospective study carried out at the Department of Head and Neck Surgery of Zhejiang Cancer Hospital between January 2016 and June 2018. The ethics protocol was obtained from the Ethics Committee of our hospital. Patients received thyroidectomies for malignant PTC. Bilateral or ipsilateral CLND was carried out for the treatment of PTC with suspicious enlarged lymph nodes on the bilateral or ipsilateral sides. Patients were excluded if they met one of the following exclusion criteria: (1) known hyperparathyroidism; (2) pregnancy and/or breast-feeding; (3) concomitant serious illnesses like chronic renal failure and hepatic insufficiency; (4) immunological diseases like arthritis and systemic lupus erythematosus; (5) diabetes mellitus; and (6) infectious diseases like HBV-hepatitis, human immunodeficiency virus, infectious atypical pneumonia, pulmonary tuberculosis, and syphilis. Written informed consents were obtained from all participants before surgery and blood sample collection.

### 2.2. Grouping Strategy

Patients were divided into comparative groups according to gender (male/female), calcemia (hypocalcemia/normocalcemia), and surgical strategies. Patients who underwent thyroidectomy with bilateral CLND (bilateral or ipsilateral cervical lymph node dissection) were assigned to the bilateral group. Patients who underwent thyroidectomy with ipsilateral CLND (with or without ipsilateral cervical lymph node dissection) were assigned to the ipsilateral group.

### 2.3. Hypocalcemia and Treatment

Normocalcemia was defined as serum Ca ≥ 2.0 mmol/l without numbness and convulsion during hospitalization. Supplementation of Ca was not needed for patients with normocalcemia. Transient hypocalcemia was defined as serum Ca <2.00 mmol/l with numbness and/or convulsion on postoperative day 1. Postoperative calcium prophylaxis was required for patients with transient hypocalcemia. Convulsive patients were treated with an intravenous administration of calcium gluconate (1 g/10 ml/day) for two days. Patients with numbness were encouraged to take routine calcium carbonate D3 granules or alfacalcidol soft capsules. Temporary hypoparathyroidism was defined as symptomatic hypocalcemia (occasional numbness and/or convulsion, with <2.0 mmol/l serum Ca) during hospitalization and at the postoperative 3-month follow-up. Asymptomatic subjects with normal Ca levels on follow-up were asked to phase out daily Ca and vitamin D supplements. Asymptomatic or symptomatic patients that had low serum PTH levels (<15.0 mmol/l) at the postoperative 6-month follow-up were diagnosed with permanent hypoparathyroidism. Daily Ca and vitamin D supplements and routine hospital follow-up were required for subjects with hypoparathyroidism.

### 2.4. Biochemical Analysis

Preoperative blood samples were collected on the morning of the operation to determine the preoperative Ca and PTH levels. The postoperative serum Ca and PTH levels were monitored on the morning of the 1^st^ (within 24 hours postsurgery), 3^rd^, 5^th^, and 7^th^ postoperative days. A low PTH level was defined as a PTH measurement <15 pg/mL within 24 hours of surgery. Patients were discharged on the 3^rd^∼7^th^ day postsurgery and were requested to return for clinical follow-up on the 1^st^, 3^rd^, 6^th^, and 12^th^ postoperative months. The serum Ca and PTH levels were measured using a Hitachi 7600 series automatic biochemical analyzer (Hitachi, Tokyo, Japan) and electrochemiluminescence system (Roche Diagnostics GmbH, Mannheim, Germany).

### 2.5. Data Collection

Biochemical characteristics including the preoperative and postoperative serum PTH and Ca levels were recorded. Demographic characteristics including age and gender; clinical characteristics such as duration of illness, tumor stage (FIGO classification), tumor diameter, node number, and lymphatic metastasis; postoperative complications (numbness and convulsion); and hypocalcemia frequency were recorded.

### 2.6. Statistical Analysis

Statistical analyses were conducted using SPSS 22.0. Dichotomous variables were described as number and percentage. Differences in dichotomous variables were analyzed using the *χ* [2] test. Quantitative data with abnormal distribution are described as median and range (Q1∼Q3). Differences in continuous variables were analyzed using the nonparametric Mann–Whitney *U* test. Logistics regression analysis was performed to identify the independent risk factors for transient hypocalcemia, with the identification of odds ratio (OR) and 95% confidence interval (CI). The area under a receiver characteristic (ROC) curve (AUC) was used to measure the relative predictability of independent factors. The sensitivity and specificity of risk factors were calculated using the Clinical Calculator 1 (http://vassarstats.net/clin1.html) with the identification of 95% CI. A *p* < 0.05 was considered to be statistically significant.

## 3. Results

### 3.1. Demographic, Clinical, and Biochemical Characteristics of Patients Included

A total of 176 patients who underwent total thyroidectomy for PTC were included (Table 1), including 155 patients with bilateral CLND and 21 patients with ipsilateral CLND, respectively. Most patients were female (77.84%, 137/176). The median age was 46.00 (17∼73) years. The median preoperative serum PTH and Ca levels were 46.00 (16.52–234.90) pg/ml and 2.35 (2.04–3.21) mmol/l, respectively. The median serum PTH and Ca level on postoperative day 1 was 18.00 (1.20–70.20) pg/ml and 2.00 (1.56–2.48) mmol/l, respectively (Table 1). Twenty-nine (16.48%) and 57 patients (32.39%) had postoperative numbness and convulsions, respectively; 51 patients (28.98%) developed transient hypocalcemia on postoperative day 1; and 5 patients (2.84%) developed temporary hypoparathyroidism within the 1^st^ month postsurgery (Table 1). Two patients (1.14%) developed permanent hypoparathyroidism during the 12-month follow-up.

### 3.2. Postoperative Recovery of Serum PTH and Ca Levels

**Figure 1:**
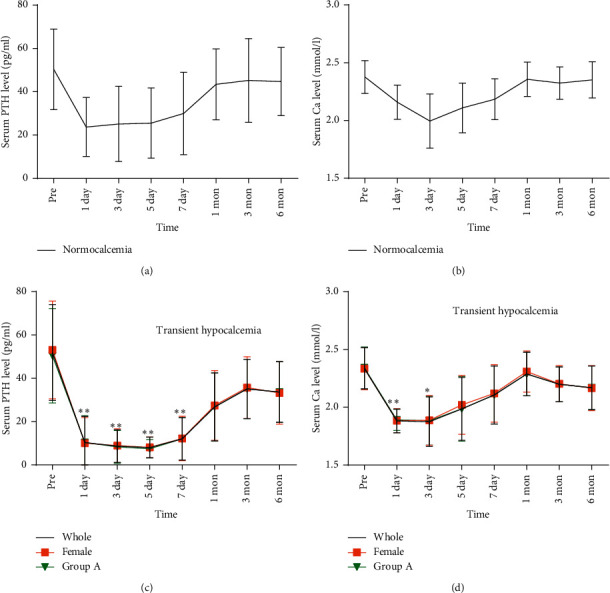
Changes in serum PTH and Ca before and after total thyroidectomy: (a) PTH and (b) Ca levels in patients who developed normocalcemia; (c) PTH and (d) Ca levels in patients who developed transient hypocalcemia. ^∗^*p* < 0.05 vs. preoperative; ^∗∗^*p* < 0.01 vs. preoperative. Pre, preoperative.

The postoperative serum PTH and Ca levels in patients with normocalcemia returned to the preoperative levels within the 1^st^ month postsurgery (Figures 1(a) and 1(b)). In patients with transient hypocalcemia, the postoperative day 1 serum PTH and Ca levels were significantly reduced (PTH from 51.89 to 10.30 pg/ml (80.15% reduction)) and Ca was decreased from 2.34 to 1.88 mmol/l (19.51%, reduction; *p* < 0.01; Figures 1(c) and 1(d)). The postoperative day 1 serum PTH level was low during the first 7 days and was at its lowest level on postoperative day 5 (Figure 1(c)). The postoperative 3-month PTH level was lower than the preoperative level. The serum Ca was at its lowest level on postoperative day 1 (Figure 1(d)) and was gradually returned to a normal level within the 1^st^ month postsurgery. The slight decrease in serum Ca level after 1 month postsurgery may be due to the phasing-out of Ca supplementation. The female gender and bilateral or ipsilateral cervical lymph node dissection did not influence the recovery of postoperative serum PTH and Ca levels.

### 3.3. Comparison between Groups

Comparison between patients with hypocalcemia (*n* = 51) and normocalcemia (*n* = 125) on postoperative day 1 demonstrated that there were significant differences in male ratio (7.84% vs. 28.57%, *p*=0.004), PTH level reduction (86.19% vs. 53.01%, *p* < 0.001), tumor diameter (1.30 vs. 0.90 cm, *p*=0.028), and preoperative serum Ca levels (2.32 vs. 2.36 mmol/l, *p*=0.034; Table 2). A significant difference was seen in the postoperative day 1 serum PTH level in patients with normocalcemia and hypocalcemia (22.06 vs. 6.35 pg/ml, *p* < 0.001).

There was no difference in the frequency of postoperative transient hypocalcemia, postoperative serum PTH and Ca levels, and the morbidity of postoperative complications between the two groups (Table 3). These findings suggested that the bilateral or ipsilateral CLND did not influence the postoperative serum PTH and Ca levels in patients who underwent thyroidectomy.

### 3.4. Identification of Independent Risk Factors for Transient Hypocalcemia on Postoperative Day 1

Univariate and multivariate logistic regression identified that PTH level reduction was an independent risk factor for transient hypocalcemia in the whole cohort (*β* = 0.043, OR = 1.044, 95% CI 1.023–1.065, *p* < 0.001), bilateral group (*β* = 0.042, OR = 1.043, 95% CI 1.022–1.064, *p* < 0.001), and female patients (*β* = 0.049, OR = 1.050, 95% CI 1.026–1.075, *p* < 0.001). Univariate regression analysis confirmed that female gender and tumor diameter were risk factors for transient hypocalcemia in the whole cohort and the bilateral group (*p* < 0.05, Table 4). Univariate and multivariate logistic regression analyses demonstrated that tumor diameter was also a risk factor for transient hypocalcemia in female patients (*p* < 0.05), but not in male patients (*p* > 0.05, Table 4). No risk factors were found in male patients.

### 3.5. Accuracy and Threshold of Predicative PTH Level Reduction

**Figure 2:**
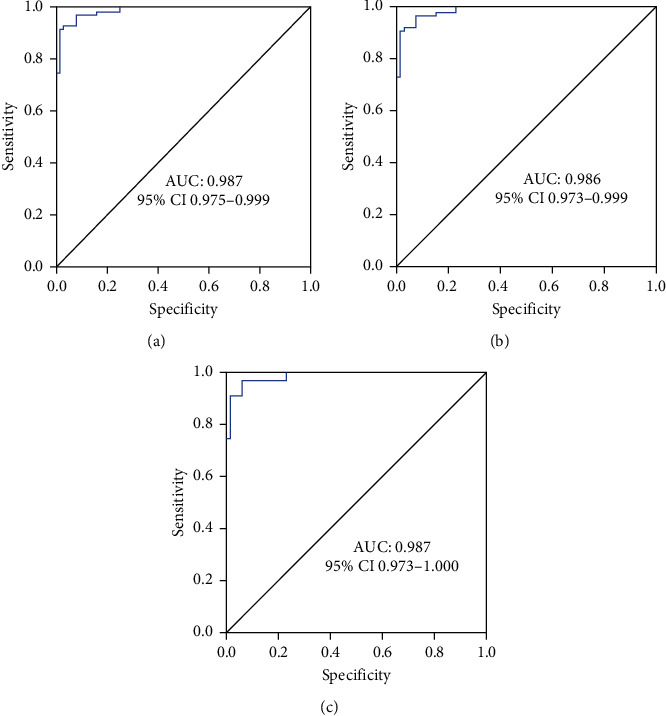
The ROC curves for various patients: (a) the ROC curve analysis of PTH reduction in predicting postoperative day 1 hypocalcemia in the whole cohort (*n* = 176); (b) the ROC curve analysis of PTH reduction in predicting postoperative day 1 hypocalcemia in patients who underwent thyroidectomy with bilateral central lymph node dissection (bilateral group, *n* = 155); (c) the ROC curve analysis of the PTH decrease in predicting postoperative day 1 hypocalcemia in female patients (*n* = 137).

Analysis of ROC curve demonstrated that the reduction in PTH level exhibited high accuracy in predicting the incidence of transient hypocalcemia in the whole cohort (AUC = 0.987, 95% CI 0.975–0.999, *p* < 0.001; Figure 2(a)), bilateral group (AUC = 0.986, 95% CI 0.973–0.9996, *p* < 0.001; Figure 2(b)), and female patients (AUC = 0.987, 95% CI 0.973–1.000, *p* < 0.001; Figure 2(c)).

The ROC curve indicated a threshold of 65.58%, 71.00%, and 71.00% PTH level reduction was reliable for predicting the incidence of transient hypocalcemia in the whole cohort, bilateral group, and female patients with a sensitivity of 53.01% (95% CI 41.80–63.94), 60.32% (95% CI 47.20–72.18), and 60.32% (95% CI 47.20–72.18) and a specificity of 92.47% (95% CI 84.60–96.67), 89.13% (95% CI 80.49–94.38), and 89.19% (95% CI 79.28–94.88; Table 5), respectively.

## 4. Discussion

Frequent monitoring of serum Ca and PTH levels after surgery is reliable for predicting postoperative hypocalcemia. Our present study illustrated that PTH level reduction, female gender, and tumor diameter were risk factors for postoperative hypocalcemia in patients who underwent total thyroidectomy with CLND. However, age, gender, and preoperative PTH and Ca levels did not show predictive features for postoperative hypocalcemia. There were differences in the thresholds of PTH level reduction for predicting the incidence of transient hypocalcemia in the whole cohort, patients with bilateral CLND, and female patients (65.58%, 71.00%, and 71.00%, respectively).

Most surgeons are interested in predicting early hypocalcemia after thyroidectomy. The early prediction of hypocalcemia may be necessary for the timely treatment and prevention of it and reduce hospital stay [15]. Some surgeons showed that patients with a <80% PTH level reduction could be safely discharged on the day of surgery [16]. Others expected but failed to predict the early discharge at 24 h posttotal thyroidectomy based on the postoperative day 1 PTH and Ca levels or delta PTH and Ca levels without considering the complications [15]. However, the morbidity of postoperative hypocalcemia is the most common cause of prolonged hospital stay after thyroidectomy [16].

The reliability of postoperative 20-min, 1-hour, 2-hour, and 3-hour PTH levels for predicting postoperative hypocalcemia has been confirmed [2, 11, 12, 16]. For instance, Reddy et al. found that the PTH level at 20 minutes after total thyroidectomy was reliable for predicting clinical hypocalcemia [12]. Early prediction of postoperative hypocalcemia is certainly desirable; however, certain surgeons have indicated that the postoperative 1-hour PTH level has equivalent reliability to the postoperative day 1 PTH levels for predicting the development of symptomatic hypocalcemia [11, 13]. As an example, Seo et al. indicated that the postoperative 1-hour and 1-day PTH levels had equivalent predictive power regarding the development of hypocalcemia [13]. We found that the serum PTH remained at stable levels within the first five days postsurgery. The low PTH level observed during hospitalization may be due to data missing from patients with normal PTH and Ca levels on postoperative day 1. The number of patients with recorded postoperative Ca levels decreased from 176 on day 1 to 101 on day 3 and 60 on day 7. However, the equivalent levels of postoperative serum PTH and Ca during the first 3 days postsurgery in patients exhibiting hypocalcemia may prove that the postoperative day 1 PTH levels are accurate enough to direct the initiation of Ca supplementation.

In our study, 28.98% (51/176) of patients displaying transient hypocalcemia on postoperative day 1 and 2 patients (1.14%) developed permanent hypoparathyroidism at the 1-year follow-up. All of these incidences were within the range of the incidence of transient and permanent hypocalcemia as previously reported [1–3, 13, 17, 18]. The differences in hypoparathyroidism incidence were attributed to the various thresholds used by the surgeons. The duration for defining permanent hypoparathyroidism ranges from 3 to 12 months postsurgery, the PTH levels range from 8.0 pg/ml to 15.0 pg/ml [13, 19–21], and serum Ca levels range from 1.9 mmol/L to 2.10 mmol/L [1, 22]. For instance, Eismontas et al. defined postoperative hypocalcemia as serum Ca level <2.10 mmol/L [22], while Sands et al. set the threshold at <1.90 mmol/L [1]. The inclusion of symptomatic or asymptomatic hypocalcemia also influences the reported incidence of hypoparathyroidism [1, 12]. We defined permanent hypoparathyroidism as symptomatic or asymptomatic in patients with a persistent low level of Ca (<2.0 mmol/l) within 6 months of surgery and transient hypoparathyroidism as symptomatic patients with a serum Ca level <2.0 mmol/l. The unification of the criteria for defining transient or permanent hypoparathyroidism may be beneficial for surgeons and for surgical management.

Our logistics analysis revealed that the PTH level reduction on postoperative day 1 was an independent risk factor for transient hypocalcemia, however not preoperative Ca or PTH levels. The significant findings in our study were that the various PTH level reduction thresholds predicted transient hypocalcemia in different cohorts. The threshold of PTH level reduction in the whole cohort, bilateral group, and female patients was 65.58%, 71.00%, and 71.00%, respectively, with moderate-high sensitivity and specificity. The 65.58% PTH level reduction threshold was equivalent to the 68.5% reported by Lecerf et al. [14]. Lecerf et al. suggested that a 68.5% PTH level reduction in patients was a more precise indicator than the postoperative PTH (19.4 ng/l) in predicting transient hypocalcemia [14]. Consistent with the reports of Seo et al., the mean relative decline (70%) in postoperative 1-hour PTH levels showed comparable reliability with the mean postoperative PTH level (10.42 pg/mL) for predicting postoperative hypocalcemia, with higher sensitivity and specificity [13]. Our present study confirmed high accuracy, sensitivity, and specificity of the PTH level reduction as well. However, the various thresholds of the PTH level reduction in the whole cohort, bilateral group, and female patients (65.58%, 71.00%, and 71.00%, respectively) illustrate that bilateral CLND and female gender are factors that should be considered when predicting transient hypocalcemia in patients who undergo total thyroidectomy for PTC.

As documented in selected literature, total thyroidectomy without CLND induced lower rates of postoperative hypocalcemia [13, 18, 23]. Various surgeons confirmed that bilateral CLND was an independent risk factor for the development of transient hypoparathyroidism [21, 23]. Zhao et al. performed a systematic review and meta-analysis of 17 studies that included 4437 patients who underwent total thyroidectomy with and without prophylactic central neck dissection for PTC [24]. The authors reported that central neck dissection certainly increased the morbidity of temporary and permanent hypocalcemia and the necessity for postoperative radioiodine ablation [24]. Due to the metastasis status of PTCs, all patients included in this study received total thyroidectomy with bilateral or ipsilateral CLND and bilateral or ipsilateral cervical lymph node dissection. Patients who underwent total thyroidectomy with bilateral and ipsilateral CLND did not show statistical differences in the morbidity of postoperative hypocalcemia. These results were consistent with that from Seo et al. [13].

The clinical and demographic parameters including diabetes, thyrotoxicosis, malignant pathology, age, female gender, and parathyroid gland number are all indicated as risk factors for asymptomatic and transient hypocalcemia [8, 9, 23]. The probability of age as a risk factor for postoperative hypocalcemia has been controversial until now [8, 15, 18, 25]. The mechanism for the association of age and female gender with postoperative hypoparathyroidism has not been investigated. Several research studies indicated the prevalence of vitamin D deficiency [8], and some specified there was not a significant association between vitamin D and hypocalcemia [26]. The association of tumor size with the incidence of postoperative hypoparathyroidism has been reported [17, 27]. The risk factor of tumor diameter in predicting postoperative transient hypocalcemia was confirmed in female patients by univariate and multivariate regression analyses, while that in the whole cohort and bilateral group was confirmed only by univariate regression analyses. These findings may indicate that additional risk factors for transient hypocalcemia should be considered in female patients receiving total thyroidectomy with bilateral or ipsilateral CLND.

## 5. Conclusion

Our present study documented that the threshold of postoperative day 1 PTH reduction was a reliable independent risk factor for predicting transient hypocalcemia in patients who underwent a total thyroidectomy with bilateral and/or ipsilateral CLND. There was no difference in the morbidity of transient hypocalcemia between patients who underwent bilateral and ipsilateral CLND. However, female patients and patients with bilateral CLND had a greater PTH reduction threshold of 71.00% than the 65.58% observed in the whole cohort. This study confirmed that female gender and tumor diameter were existing independent risk factors for postoperative transient hypocalcemia in total thyroidectomy, while no risk factors were identified in male patients. Female patients with large tumor size and >71.00% PTH reduction on postoperative day 1 posttotal thyroidectomy with CLND had a high risk of transient hypocalcemia.

## Figures and Tables

**Table 1:** Demographic, clinical, and biochemical characteristics of the 176 patients with papillary thyroid carcinoma undergoing radical operation.

Characteristic	Value
Gender (male/female)	39/137
Age (year, median, range)	46.00 (17–73)
Tumor diameter (cm, median, range)	1.00 (0.20–5.00)
Duration of illness (day, median, range)	60 (1–2920)
Node number (single/multi/NA)	46/125/5
Total thyroidectomy (radical operation)	
Bilateral CLND	108
Ipsilateral CLND	13
Bilateral CLND + bilateral cervical LND	12
Bilateral CLND + ipsilateral cervical LND	35
Ipsilateral CLND + ipsilateral cervical LND	8
Pathology	
Pathologic N (N0/N1/N2/N3)	65/111/0/0
Pathologic T (T1/T2/T3/T4/)	132/16/28/0
Pathology stage (I/II/III/IV)	147/22/4/3
Preoperative serum PTH (pg/ml, median, range)	46.00 (16.52–234.9)
Preoperative serum Ca (mmol/l, median, range)	2.35 (2.04–3.21)
POD1 serum PTH (pg/ml, median, range)	18.00 (1.20–70.20)
POD1 serum Ca (mmol/l, median, range)	2.00 (1.56–2.48)
Postoperative complications	
Convulsion	57
Numbness	29
None	90
Hypocalcemia (male/female)	
POD1 (*n* = 176)	4/51
POD3 (*n* = 101)	8/51
POD5 (*n* = 71)	4/23
POD7 (*n* = 60)	2/11
POD30 (*n* = 100)	1/4
POD90 (*n* = 74)	0/4
POD180 (*n* = 57)	0/2
POD365 (*n* = 35)	0/1

POD1/3/5/7/30/90/180/365, postoperative day 1, 3, 5, 7, 30, 90, 180, and 365. CLND, central lymph node dissection; LND, lymph node dissection.

**Table 2:** The comparison between patients who developed hypocalcemia and normocalcemia on postoperative day 1.

Variables	Hypocalcemia	Normocalcemia	*p*
Gender (male)	7.84% (4/51)	28.57% (35/125)	0.004^*a*^
Age (year)	45.00 (35∼53)	47.00 (37.5∼53)	0.841^b^
PTH reduction (%)	86.19 (77.92∼91.17)	53.01 (33.26∼75.58)	<0.001^*b*^
Tumor diameter (cm)	1.30 (0.70∼2.25)	0.90 (0.60∼1.50)	0.028^*b*^
Tumor grade	1 (1∼4)	1 (1∼3)	0.405^b^
Preoperative serum PTH (pg/ml)	44.90 (37.12∼64.38)	46.18 (37.56∼58.43)	0.893^b^
Preoperative serum Ca (mmol/l)	2.32 (2.23∼2.41)	2.36 (2.29∼2.46)	0.034^*b*^
POD1 serum PTH (pg/ml)	6.35 (4.00∼11.51)	22.06 (13.18∼31.53)	<0.001^*b*^
POD1 serum Ca (mmol/l)	1.91 (1.84∼1.96)	2.15 (2.05∼2.25)	<0.001^*b*^
Postoperative complications			<0.001^*a*^
Convulsion	64.71% (33/51)	19.20% (24/125)	
Numbness	35.19% (18/51)	9.60% (12/125)	

POD1, postoperative day 1. ^a^Difference between groups is tested by the *χ* [2] test; ^b^difference between groups is tested by the Mann–Whitney *U* test.

**Table 3:** The impact of surgical methods on the frequency of postoperative hypocalcemia and serum hormone level.

Variable	Bilateral group	Ipsilateral group	*p*
Age (year)	45.00 (37∼53)	48.00 (41∼58)	0.433^b^
POD1 hypocalcemia	30.97% (48/155)	14.29% (3/21)	0.132^a^
Gender (male)	5/48	0/3	1.000^a^
PTH reduction (%)	62.20 (40.70∼83.93)	70.35 (19.71∼87.49)	0.943^b^
Postoperative complications			0.645^a^
Convulsion	30.97% (48/155)	42.86% (9/21)	0.319^a^
Numbness	18.71% (29/155)	0 (0/21)	*0.030 * ^*a*^
None	50.32% (78/155)	57.14% (12/21)	0.557^a^
Preoperative serum PTH (pg/ml)	46.43 (37.6∼59.67)	42.34 (32.71∼66.11)	0.599^b^
Preoperative serum Ca (mmol/l)	2.34 (2.27∼2.45)	2.36 (2.33∼2.45)	0.310^b^

POD1/7/30/90/180, postoperative day 1, 7, 30, 90, and 180. ^a^Difference between groups is tested by the *χ* [2] test; ^b^difference between groups is tested by the Mann–Whitney *U* test.

**Table 4:** The risk factors for the transient hypocalcemia on postoperative day 1.

Variable	Univariate			Multivariate		
	*β*	OR (95% CI)	*p*	*β*	OR (95% CI)	*p*
The whole cohort (*n* = 176)						
Gender	1.268	3.555 (1.302–9.703)	*0.013*	1.160	3.189 (0.924–11.007)	0.067
Age (year)	−0.017	0.983 (0.954–1.012)	0.251	−0.001	0.999 (0.961–1.039)	0.957
PTH reduction (%)	0.047	1.048 (1.028–1.068)	*<0.001*	0.043	1.044 (1.023–1.065)	*<0.001*
Preoperative PTH (pg/ml)	0.002	1.002 (0.958–1.019)	0.849	−0.010	0.990 (0.969–1.011)	0.337
Preoperative serum Ca (mmol/L)	−1.671	0.188 (0.015–2.295)	0.190	−3.023	0.049 (0.001–1.786)	0.100
Tumor diameter (cm)	0.410	1.507 (1.059–2.145)	*0.023*	0.447	1.564 (0.981–2.496)	0.060
Surgery	0.952	2.590 (0.725–9.261)	0.143	1.002	2.722 (0.518–14.318)	0.237

Bilateral group (*n* = 125)						
Gender	1.235	3.440 (1.243–9.523)	*0.017*	1.155	3.176 (0.907–11.113)	0.071
Age (year)	−0.015	0.985 (0.955–1.015)	0.320	0.003	1.003 (0.964–1.044)	0.883
PTH reduction (%)	0.047	1.048 (1.028–1.069)	*<0.001*	0.042	1.043 (1.022–1.064)	*<0.001*
Preoperative PTH (pg/ml)	−0.005	0.995 (0.977–1.013)	0.562	−0.018	0.982 (0.960–1.004)	0.114
Preoperative serum Ca (mmol/L)	−1.123	0.325 (0.029–3.595)	0.360	−0.3024	0.049 (0.001–1.920)	0.107
Tumor diameter (cm)	0.389	1.476 (1.032–2.111)	*0.033*	0.465	1.592 (0.992–2.554)	0.054

Female patients (*n* = 137)						
Age (year)	−0.016	0.984 (0.953–1.016)	0.316	−0.001	0.999 (0.958–1.042)	0.964
PTH reduction (%)	0.051	1.053 (1.030–1.076)	*<0.001*	0.049	1.050 (1.026–1.075)	*<0.001*
Preoperative PTH (pg/ml)	0.004	1.004 (0.987–1.022)	0.643	−0.006	0.994 (0.972–1.016)	0.582
Preoperative serum Ca (mmol/L)	−1.784	0.168 (0.012–2.278)	0.180	−3.569	0.028 (0.001–1.366)	0.071
Tumor diameter (cm)	0.446	1.563 (1.070–2.283)	*0.021*	0.499	1.647 (1.003–2.704)	*0.049*

Male patients (*n* = 39)						
Age (year)	−0.024	0.976 (0.900–1.059)	0.558	−0.012	0.988 (0.869–1.123)	0.853
PTH reduction (%)	0.027	1.028 (0.988–1.069)	0.171	0.023	1.023 (0.981–1.068)	0.285
Preoperative PTH (pg/ml)	−0.028	0.973 (0.909–1.041)	0.424	−0.051	0.951 (0.867–1.043)	0.284
Preoperative serum Ca (mmol/L)	0.104	1.019 (0.000–31.581)	0.984	−0.280	0.756 (0.000–12.050)	0.964
Tumor diameter (cm)	0.120	1.127 (0.364–3.489)	0.836	0.194	1.215 (0.242–6.100)	0.813

**Table 5:** The accuracy of PTH reduction in predicting hypocalcemia and normocalcemia in the whole cohort, bilateral group, and female patients.

Variables	Hypocalcemia	Normocalcemia
Whole cohort^a^		
PTH reduction ≥ 65.58%	44 (TP)	39 (FP)
PTH reduction < 65.58%	7 (FN)	86 (TN)

Bilateral group^b^		
PTH reduction ≥ 71.00%	38 (TP)	25 (FP)
PTH reduction < 71.00%	10 (FN)	82 (TN)

Female patients^c^		
PTH reduction ≥ 71.00%	38 (TP)	25 (FP)
PTH reduction < 71.00%	8 (FN)	66 (TN)

^a^Sensitivity, 53.01% (95% CI 41.80–63.94); specificity, 92.47% (95% CI 84.60–96.67); positive predictive value, 86.27% (95% CI 73.13–93.85); negative predictive value, 68.80% (95% CI 59.81–76.62). ^b^Sensitivity, 60.32% (95% CI 47.20–72.18); specificity, 89.13% (95% CI 80.49–94.38); positive predictive value, 79.17% (95% CI 64.60–89.04); negative predictive value, 76.64% (95% CI 67.28–84.04). ^c^Sensitivity, 60.32% (95% CI 47.20–72.18); specificity, 89.19% (95% CI 79.28–94.88); positive predictive value, 82.61% (95% CI 68.05–91.68); negative predictive value, 72.53% (95% CI 62.00–81.12). FN, false negative; FP, false positive; TN, true negative; TP, true positive.

## Data Availability

All data generated or analyzed during this study are included in this published article. The original data are available from the authors upon request.

## Additional Points

(1) 28.98% patients who underwent total thyroidectomy developed transient hypocalcemia. (2) A 65.58% PTH level reduction was a risk factor for posttotal thyroidectomy transient hypocalcemia. (3) A 71.00% PTH level reduction was a risk factor for transient hypocalcemia in female patients. (4) Hypocalcemia incidence was not different between bilateral and ipsilateral CLNDs.

## Supplementary Materials

Supplementary Materials Table S1: the impact of surgical methods on serum hormone level.
