# Supplementary material for: Parathyroid Hormone Reduction Predicts Transient Hypocalcemia after Total Thyroidectomy: A Single-Center Prospective Study
Source: Int J Endocrinol. 2020 Aug 1;2020:7189857. doi: 10.1155/2020/7189857 (PMC7416261; doi:10.1155/2020/7189857)
Supplement: Supplementary Materials — Table S1: the impact of surgical methods on serum hormone level. [file 7189857.f1.docx]

**Table S1**. The impact of surgical methods on serum hormone level.

| Variable | Bilateral group | Ipsilateral group | P |
| --- | --- | --- | --- |
| POD7 serum PTH (pg/ml) | 12.60(8.91~35.28) | 23.06 | 0.607 ^b^ |
| POD7 serum Ca (mmol/l) | 2.22(2.01~2.30) | 2.12 | 0.583 ^b^ |
| POD30 serum PTH (pg/ml) | 33.14(20.72~49.39) | 47.15(23.22~51.90) | 0.415 ^b^ |
| POD30 serum Ca (mmol/l) | 2.35(2.23~2.44) | 2.42(2.22~2.45) | 0.313 ^b^ |
| POD90 serum PTH (pg/ml) | 39.65(29.86~51.33) | 39.86(28.30~63.45) | 0.793 ^b^ |
| POD90 serum Ca (mmol/l) | 2.31(2.22~2.39) | 2.33(2.26~2.35) | 0.938 ^b^ |
| POD180 serum PTH (pg/ml) | 39.49(29.63~50.84) | 36.17(17.94~73.64) | 0.817 ^b^ |
| POD180 serum Ca (mmol/l) | 2.32(2.23~2.39) | 2.28(2.19~2.31) | 0.226 ^b^ |

POD1/7/30/90/180, postoperative day 1, 7, 30, 90 and 180. b, difference between groups is tested by Mann-Whitney U test.
